# Integrating service user participation in mental health care: what will it take?

**DOI:** 10.5334/ijic.1992

**Published:** 2015-03-04

**Authors:** Sharon Lawn

**Affiliations:** Flinders Human Behaviour & Health Research Unit, Flinders University, Adelaide, SA, Australia

**Keywords:** service user participation, mental health care, definition of integrated care

## Abstract

Participation in mental health care poses many challenges for mental health service users and service providers. Consideration of these issues for improving the integration of service user participation in mental health care can help to inform integrated care within health care systems, broadly. This paper argues for practicing greater empathy and teaching it, stigma reduction, changing what we measure, valuing the intrinsic aspects of care more, employing more people with lived experience within mental health services, raising the visibility of service users as leaders and our teachers within services and redefining integrated care from the service user perspective.

Participation in mental health can mean individually focused collaborative, respectful inclusion of mental health service users in care decisions within their interpersonal encounters with service providers and systems. But it can also mean engagement with the notion of having a mental illness and seeking or accepting support. It can also mean participation at service and system level as peer workers and as advocates for policy and practice change. This paper represents an invited presentation at the World Conference on Integrated Care held in Sydney, Australia, in November 2014. It focuses largely on the first of these forms of participation, individual participation in care, with some brief comments on peer participation. A note on language: various terms are used throughout the literature (consumer, patient, client, service user). The preferred term ‘service user’ is used here to mean the person who receives health services. The ideas raised here will hopefully resonate with integrating health care service user participation more broadly. The subtitle of this paper (What will it take?) is deliberate because I argue that we are not there yet; that there is still much to do to achieve useful, meaningful and true service user participation in mental health.

The term ‘integrated care’ is used here to mean, ‘A concern to improve patient experience and achieve greater efficiency and value from health delivery systems. The aim is to address fragmentation in patient services, and enable better coordinated and more continuous care’ [1, p. 3]. Integrated care is therefore:

An organising principle for care delivery that aims to improve patient care and experience through improved coordination … Integration is: the combined set of methods, processes and models that seek to bring this about. It requires that those involved with planning, financing and providing services have a shared vision … and ensure that the patient's perspective remains a central organising principle throughout. [1, p. 7]

Kodner and Spreeuwenberg [2] emphasise that it should therefore improve quality of care, user experience of care and its cost-effectiveness. It is an interesting definition because the service user is essentially the subject or object to which methods, processes and models are administered. Some might say it risks being delivered as well-intentioned, yet paternalistic care. It depends on how participation is understood and operationalised.

Notions of client-hood and citizenship, central to recovery, also assist in understanding service user participation in mental health. Borg and Davidson [3] argue that a central feature of recovery-based practice for people with mental illness is that they are able to exercise rights and experience membership of a community. Recovery involves the person's capacity for agency; how they are enabled to maximise a positive sense of self as a citizen, and minimise threats to agency by what Fisher [4] describes as ‘being done to’ within systems of care.

Mental health provides the sharp focused lens on issues that are important across all areas of health care. In mental health care, we see not only the stark view of how people treat each other, their enormous capacity for compassion but also negative aspects of what is done to people by services, and the blunt impact of stigmatising culture and community values; sometimes from within mental health services themselves. This is because there is a fundamental tension within mental health care. It is one of the few health care areas where people can be detained against their will, and where services can enforce treatment in order to ‘care’ for them. Some parts of dementia care arguably offer the only parallel system to this.

So what does all this mean for consumer participation in mental health? Recently, I led a project in which involved in-depth qualitative interviews with people on Community Treatment Orders (legally enforced treatment in the community because of being assessed as a danger to themselves or others) and mental health professionals who work with them. We found that, for many worker participants, their interactions with service users on Community Treatment Orders involved being benevolent towards them, acting virtuously towards them and striving for ways to soften the coercive stick inherent in the Community Treatment Order process through attempts to empathise with service users’ experience. For almost all service user participants, the Community Treatment Order experience was understood as morally framed, perceiving that they were being punished for being bad, being seen as untrustworthy and having faults to be corrected via coercive practices by services that worked against their full engagement in the recovery process.

Gault, Gallagher and Chambers [5] describe the person who is subject to Community Treatment Order legislation as ‘a discredited identity’ who, in their attempts to regain some control, resort to ‘playing the game’ of appearing to be compliant because appearing to behave in a certain manner keeps workers off their back. Clarke, in his critique of UK social policy, describes mental health service users in general as, “objects of intensified surveillance, criminalization and incarceration” [6, p. 458]. Sawyer argues that the growing focus on risk by mental health services has altered the workers’ role and, ‘diminished the significance and legitimacy of therapeutic responses” [7, p. 287]. She describes mental health workers as, ‘psychiatric risk managers’ in a community that has become one of containment and ensuring security. This represents a complex and contradictory paradox in which caring, engaging and building rapport whilst simultaneously policing become complicated.

So where do this leave service user participant in mental health and indeed integrated care more broadly? What can be done differently? Denhov and Topor [8], conducting interviews with 71 people with mental illness in Sweden, found that trust is central to the relationship and takes time. Characteristics of helpful professional were that they were ‘nice, friendly, humane, attentive, obliging, helpful, patient, genuinely interested and genuinely involved’ [8, p. 420]. Participants’ perception of the professionals’ underlying attitude towards them was pivotal to the relationships being a trusting one. Bracken and colleagues [9] argue that the development of meaningful, non-judgmental relationships are as much involved in mental health recovery as the therapies and psychiatric medications used to treat mental illness, calling for greater empathy for service users’ experiences. This requires the professional's willingness to suspend judgement and appreciate the service user's perspective [10]. Rogers called it ‘unconditional positive regard’ [11].

Shared decision-making and self-management are ways that service user participation are promoted within health services. However, these concepts place slightly different emphasis on the role of information and expertise. Shared decision-making is about supporting people ‘to understand evidence-based information about treatment probabilities and risk regarding a specific decision. This assumes a limited set of options for available decisions and actions, arguably laid out by the health professional for the service user to choose from. Self-management is about supporting the person to incorporate evidence-based health information into their daily lives, i.e. taking from it and blending it with their own lived experience expertise. Also underpinning service user participation is person-centred care [12]. The arguments for person-centred care range from it being ‘the right thing to do’; of intrinsic benefit for people feeling respected, valued and involved; hence, the term in mental health – ‘Nothing about me, with me’. Further arguments are that more engaged and informed service users mean better outcomes and cost-effectiveness. These latter arguments tend to dominate implementation goals within services and can lead to intrinsic benefits being overlooked as soft measures. They can also lead to an emphasis on service users being urged to become ‘responsible’ regardless of their differing capacities to do so and for some to be blamed as a consequence of failing to become ‘responsibilised citizens’ [6].

At this point you might be asking, ‘What are the benefits of service user involvement in mental health services?’

Benefits noted in the literature include: more effective partnerships of care between service users, carers and workers; better understanding by service providers of the experience of mental illness for service users and their families; better targeted services based on identified needs; greater recognition of the effectiveness of particular interventions; and greater service user empowerment, confidence and feeling valued, which leads to enhanced quality of life [13]. Despite these benefits, service user involvement in mental health has been a slow journey. A study in south-east England sought service users’ views about level of involvement [13] found a number of areas needing improvement. Two-thirds of participants had not been asked about or given a choice in their hospital care and treatment. Approximately one-third of participants did not feel encouraged by professionals to say what their aims were or given a choice for care and treatment in the community.

Glasby and Lester [14] identified a number of barriers to service user participation. These include a lack of information provision to them, whereas better health literacy would lead to more informed choices. Perceptions held by health professionals and organisations included that service user involvement is a more expensive investment and concern that the mechanisms for care delivery are tokenistic. There were concerns about representativeness of service users on committees as an excuse for avoiding user involvement. Double standards were also evident: the views of those who say challenging things can be discounted, discredited as non-representative, or even interpreted as the service user being unwell. Tensions in care, especially in the context of compulsory treatment, were also apparent; the sense that as health professionals, ‘we know best’ and a lack of acknowledgement by workers of service users’ ‘expertise’.

The above perceptions are concerning and need to be addressed with evidence for the value of service user participation. However, a significant issue is the lack of robust measurement of impact and poor quality of reporting the impact of service user involvement [15]. Mockford et al. [15] suggest three general types of impact that could be measured more effectively: (1) Impact on service planning and development (e.g. type of services, design of buildings, co-design of resources); (2) Impact on information development and dissemination (e.g. patient information leaflets, contributing to staff training sessions); and (3) Impact on attitudes of service users and providers.

Mental health peer workers are experts by their lived experience of mental illness. There are three fundamental arguments for their value as participants in mental health care. First, they are the evidence for recovery, challenging perceptions of hopelessness and chronicity held by people with mental illness, carers and mental health professionals. They also blur the boundary between sickness and health, challenging stigma and challenging staff to reflect on their practice. They can help each other, challenging the assumption that people with mental illness primarily and always require ‘professional’ help. The focus of peer input is on finding meaning, not diagnostic criteria, and this has benefits for recovery in the longer term [16].

However, there are a number of challenges to peer workers ‘staying true’ to the peer philosophy, covered only briefly here. There are challenges in providing support without succumbing to the issue of ‘othering’ the person or becoming mere agents of professionals’ view of what the person needs or ‘should’ do or be. ‘Professionalisation’ of the peer workforce is an issue in some contexts and for some individuals, dependent on their location, the service culture of the setting in which they work, the level of independence the service gives to peers and clinical pressures within the setting. As a consequence of the range of these challenges, ‘colonisation’ of the peer role can occur by clinical services [17].

There are a number of legitimate reasons for why these issues might arise. There are challenges in not losing the very qualities that make peer support unique and of value, and balancing this within training to prepare peers for organisational working and using their peer qualities. Supporting peers to understand how to stand alongside the very big shadow cast by organisations and systems of health care requires ongoing support and mentorship. Otherwise, we throw people in the deep end with few tools other than their lived experience. Current systems of care are set up with little capacity to navigate the peer role. This can lead to unhelpful boundary and turf debates due to the lack of a comprehensive model for the role like other more traditional disciplines, given it is ‘the thing itself’, the lived experience that is central to the peer role.

So, what will it take to integrate service user participation in mental health? Perhaps it will take the community ensuring participation in citizenship for people with mental illness. We can do this by practicing greater empathy and teaching it [18] to all people working with people with mental illness, and working to reduce stigma, starting with ourselves [19]. We can change what we measure, and value the intrinsic aspects of care more [15]. We can employ more people with lived experience of mental illness within health services and let them use that experience, because they are there already, but may not have the support of their organisations to declare and overtly use this experience. Raising the visibility of service users as leaders in key roles in the mental health sector would also help to send a clearer message to influence service culture. We can redefine integrated care from the service user perspective [20] rather than from the system perspective which risks maintaining the service user as the object of care rather than as participant. Finally, acknowledging service users as our teachers would cast them in a more valued and empowered light and benefit integrated care for us all.

## About the author

Sharon Lawn, PhD, is Associate Professor and Executive member for the Flinders Human Behaviour and Health Research Unit, within the Department of Psychiatry at Flinders University in South Australia. Throughout her career she has worked across the boundaries of practice, policy, advocacy and research. Her primary fields of interest are mental health systems of care, smoking and mental illness, consumer and carer perspectives, and chronic condition management and self-management approaches; in particular, the ethnographic experiences and culture of care systems for service users and service providers. Sharon has worked in several projects, including national audits of smoke-free policy for England and for Australia, provision of the evidence brief for the Australian National Chronic Disease Strategy, chronic condition management Capabilities for the National Primary Care Workforce, and the Coordinated Veterans Care Program. Sharon has won awards nationally for her work in mental health and is very engaged in advocacy in this area. Sharon has published many articles in Australia and abroad.
